# Controlled hypotension during neuraxial anesthesia is not associated with increased odds of in-hospital common severe medical complications in patients undergoing elective primary total hip arthroplasty – A retrospective case control study

**DOI:** 10.1371/journal.pone.0248419

**Published:** 2021-04-01

**Authors:** Jiabin Liu, Haoyan Zhong, Danya DeMeo, Huong Do, Meghan Kirksey, Alejandro Gonzalez Della Valle, Jacques YaDeau

**Affiliations:** 1 Department of Anesthesiology, Critical Care & Pain Management, Hospital for Special Surgery, Weill Cornell Medical Center, New York, NY, United States of America; 2 Clinical Data Core, Hospital for Special Surgery, New York, NY, United States of America; 3 Department of Orthopedic Surgery, Hospital for Special Surgery, Weill Cornell Medical Center, New York, NY, United States of America; University of Pennsylvania, UNITED STATES

## Abstract

**Introduction:**

The use of controlled hypotension during neuraxial anesthesia for joint arthroplasty is controversial. We conducted a large institutional database analysis to assess common in-hospital complications and mortality of patients undergoing primary total hip arthroplasty (THA) under controlled hypotension and neuraxial anesthesia.

**Methods:**

We conducted a large retrospective case control study of 11,292 patients who underwent primary THA using neuraxial anesthesia between March 2016 and May 2019 in a single institution devoted to musculoskeletal care. The degree and duration of various mean arterial pressure (MAP) thresholds were analyzed for adjusted odds ratios with composite common severe complications (in-hospital myocardial infarction, stroke, and/or acute kidney injury) as the primary outcome.

**Results:**

Sixty-eight patients developed common severe complications (0.60%). Patients with complications were older (median age 75.6 vs 64.0 years) and had a higher American Society of Anesthesiologists (ASA) classification (45.6% vs 17.6% ASA III). The duration of hypotension at various MAP thresholds (45 to 70 mm Hg) was not associated with increasing odds of common severe medical complications.

**Conclusions:**

Controlled hypotension (ranging from 45 to 70 mmHg) for a moderate duration during neuraxial anesthesia was not associated with increased odds of common severe complications (myocardial infarction, stroke, and/or acute kidney injury) among patients receiving neuraxial anesthesia for elective THA.

## Introduction

Neuraxial anesthesia has been associated with lower risk of transfusion and decreased short-term complications including venous thromboembolism, decreased length of stay, and facilitating home discharge following total hip arthroplasty (THA) [1–4]. Neuraxial anesthesia is preferred by over 70% of board-certified arthroplasty surgeon members of the American Association of Hip and Knee Surgeons [5]. During conventional neuraxial anesthesia, blood pressure is maintained by balancing intravascular volume status, peripheral vascular resistance, and cardiac contractility with intravenous fluid and/or vasoactive medication administration. Uncontrolled intraoperative hypotension precipitated by anesthetic agents, surgical blood loss, or physiologic insults such as micro emboli can result in major complications, such as myocardial infarction, stroke, neurocognitive dysfunction, acute kidney injury, and mortality [6–9]. The risk of such adverse outcomes is related to the degree and duration of hypotension and hypo-perfusion. Consensus statements have been published regarding aggressive intraoperative blood pressure management during elective surgery [8], recommending that MAP may not be lowered more than 20~30% of the baseline during anesthesia.

In contrast, controlled hypotension during neuraxial anesthesia for joint arthroplasty is usually associated with different pathophysiology. However, its application in orthopedic surgery remains controversial [10]. Our institution has been practicing neuraxial anesthesia-induced intraoperative controlled hypotension (IOCH) for more than 30 years [4] with lower blood pressure thresholds in each given case determined at the discretion of the surgeon and anesthesiologist. Under these conditions, hypotension is not secondary to excessive surgical bleeding, anesthetic agent overdose, or other misadventure. Although these patients have low blood pressure, the cardiac output is usually augmented via the positive vasoactive effect of an epinephrine infusion, thereby generating a high flow, low pressure state [11].

The use of IOCH in joint arthroplasty made a significant contribution to the improvement of surgical outcomes, especially during the early practice of joint arthroplasty [4, 12]. With the improvement of surgical techniques, less cementing, and use of tranexamic acid, our institutional practice guideline has largely ceased to pursue extremely low blood pressure for surgical field optimization. However, we continue to find lowering of blood pressure during hip arthroplasty surgery to be useful for minimization of bleeding in the surgical field, and therefore mild to moderate low blood pressure is still a common practice at our institution. IOCH has substantial advantages for the surgical team, as the orthopaedic surgeon can proceed with surgical exposure expeditiously through a bloodless field, with better surgical exposure, shorter surgical time, and less intraoperative blood loss. Using a combination of neuraxial anesthesia and IOCH, our institution has consistently demonstrated serious medical complication and mortality rates far below the national average [13, 14]. However, the impact of prior studies has been limited by relatively small sample size. The goal of this study was to assess the risk of common perioperative complications in a large cohort of patients undergoing THA with neuraxial anesthesia and different levels of IOCH. We hypothesize that mild to moderate IOCH does not increase patients’ risk of common perioperative complications.

## Material and methods

This retrospective case control study was approved by the Hospital for Special Surgery institutional review board (IRB# 2018–2235). The study followed STROBE standards [15]. All data were fully anonymized after the initial data extraction and before further data analysis, and the IRB waived the requirement for informed consent.

**Fig 1:**
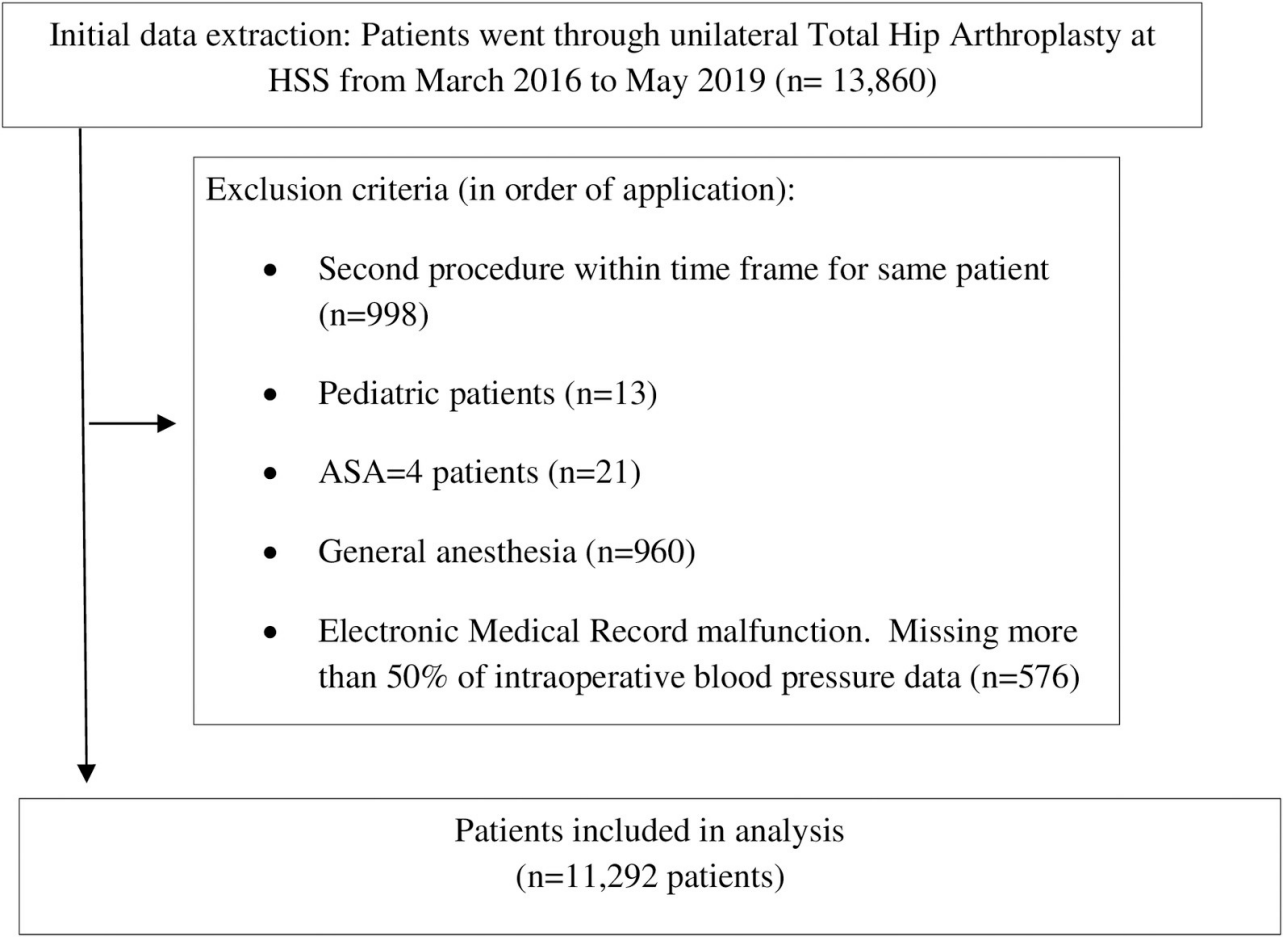
Inclusion and exclusion criteria of patient cohort selection.

All 13,860 patients who underwent primary, elective, unilateral THA at our institution between March 11, 2016 and May 17, 2019 were identified using the institutional electronic medical record platform (Epic, Verona, USA). The inclusion criteria included age older than 18, ASA 1–3, received spinal/epidural anesthesia. Patients were subsequently excluded if they underwent a second independent procedure within the same study period (n = 998), if they were younger than 18 years at the time of surgery (n = 13), if they had an ASA class of 4 (n = 21), if they received general anesthesia (n = 960), or if there were >50% of intraoperative blood pressure data missing (n = 576). The final cohort included 11,292 patients (Fig 1).

### Intraoperative management

The most common anesthetic for THA in our institution is a combination of neuraxial anesthesia and intravenous sedation. Neuraxial anesthesia was delivered via spinal anesthesia (3600 patients), or epidural with/without spinal anesthesia (7692 patients). Patients often received arterial-line monitor during the procedure (83.9%) to facilitate management of IOCH. The mean arterial pressure (MAP) is managed by balancing the effects of neuraxial anesthesia induced sympathectomy, intravenous fluid volume, and use of inotropes (such as epinephrine infusion and ephedrine). Such approach will provide low pressure low vascular resistance with preserved or even increased cardiac output status [4]. The targeted MAP threshold is typically between 50 and 60 mmHg adjusted based on patient comorbidities.

### Intraoperative arterial pressure

Intraoperative MAP data were defined as blood pressure measurements collected from the anesthesia start time to anesthesia end time. The following standards were applied to blood pressure data cleaning process:

1. If invasive monitoring via arterial line existed, we used MAP measurements at 1-minute intervals. There were 9573 patients with invasive arterial line monitor (84.8%). If arterial line monitoring was not used, regular noninvasive intraoperative blood pressure measurements were used to calculate MAP at 5-minute intervals.
2. Blood pressure recording before anesthesia start time and blood pressure recording after anesthesia end time were removed. MAP<20 mmHg or >200mmHg time points were considered as artificial noise and removed (~1% of the total blood pressure recording data).
3. Time points at which the MAP value was more than 50% different from the previous and subsequent MAP values, were deleted with the assumption of artifact.
4. For invasive monitoring via arterial line recording, if no MAP was recorded, but the time point 1 minute before and 1 minute after were recorded, then the missing MAP value would be recoded using the previous time point value. Similar for noninvasive measurement, if no MAP was recorded, but the time point 5 minutes before and 5 minutes after were recorded, then the missing MAP value will be recoded using the previous time point value. If a MAP value was missing longer than the specified time above, then it would be kept as missing.
5. After the above 4 steps, patients whose electronic medical record MAP data were missing over 50% of total anesthesia time were excluded (n = 576). Although there were handwritten paper records available likely, we elected to remove these patients.

### Outcomes and exposures

Postoperative complications during hospitalization and transferring care to New York Presbyterian hospital were identified from electronic records using the discharge diagnoses described by the International Classification of Disease, Tenth Revision, Clinical Modification (ICD-10-CM). Thirty-day mortality data were obtained from institutional quality control board as part of Adult Reconstruction and Joint Replacement quality control requirement. All identified complications were manually confirmed via chart review. Given the small number of complications of interest, and in order to better capture the outcome of interest, we chose a binary any-versus-none collapsed composite outcome as primary outcome [16]. The composite common severe complications of interest were in-hospital myocardial infarction, stroke, and/or acute kidney injury. If any of the common severe complications of interest was identified, then it will be marked as an event. All common severe complications and 30-day mortality were listed in Table 1.

**Table 1:** List of complications among 11,292 total hip arthroplasty patients.

	Count
Components of primary outcome: Acute renal failure	51
Myocardial Infarction	16
Stroke	2
In-hospital mortality	0
30-day mortality	3

The primary exposure of interest was total time of IOCH. For each patient, the duration of IOCH was defined as the total minutes during which MAP fell below a predetermined threshold, such as MAP < 60 mmHg. A range of thresholds (45–70 mmHg) were tested in sensitivity analysis.

### Statistical analysis

Statistical plans were finalized before analysis. Categorical variables were reported as frequency and percentage and analyzed using Chi Square or Fisher exact test across common complication status. Continuous variables were reported as median and interquartile range and analyzed using Mann-Whitney U test across common complication status. Multiple logistic regression models were used to determine adjusted odds ratios (OR) and 95% confidence intervals. Firth’s penalized likelihood was used to reducing small-sample bias in maximum likelihood estimation in the model. The covariables included in the regression model were statistically significant based on univariate analysis by complications of interest. Therefore, the covariables included in the model were age, sex, and ASA classification. IOCH duration and types of anesthesia were forced into the model irrelevant to the univariate analysis output based on the authors’ interest. We also conducted sensitivity analyses with MAP threshold from 45 to 70 mmHg at 1 mmHg interval adjusting for the same covariables in the same primary analysis model. An area under the curve was generated for each logistic regression model to assess the model ability to classify composite common severe complication status. We electively conducted the analysis with broader MAP threshold above 60s mmHg to be more inclusive. All analyses were performed with SAS Version 9.4 (SAS Institute, Cary, NC). All P-values reported were from two-tailed tests. A p value <0.01 was determined significant.

## Results

Sixty eight of 11,292 patients (0.60%) developed common severe complications during the period of study (Table 1). The common severe complications included myocardial infarction in 16 patients, stroke in 2 patients, and acute kidney injury in 51 patients. There were no in-hospital deaths, but three patients (0.027%) died within 30 days of surgery after discharge. None of these three mortality patients exhibited myocardial infarction, stroke, acute kidney injury, or other known complications before being discharged. The exact reasons of death were still under investigation till the drafting of this manuscript. We elected to not include these three mortality patients in the complication group due to small number of events, unknown etiology, and the fact that there was no systematic follow up of patients after discharge. There were 6 patients requiring transfer to another healthcare facility for specialty care due to medical reasons beyond our focus (including 1 seizure, 1 retinal vein occlusion, 1 vasospasm induced stroke like symptom, 1 pulmonary embolism, 1 resistant atrial fibrillization requiring cardioversion/ablation, and 1 perforated diverticulitis). We elected to include these patients in the control group to remain focused on the common severe complications.

Patients who developed common severe complications were older (75.6 [63.1, 81.6] vs 64.0 [57.0, 71.5] yrs, p<0.0001), were more likely non-white (23.5% vs 10.9%, p = 0.0005), had a higher ASA class (45.6% vs 17.6% ASA III, p<0.0001), and a longer hospitalization 3 [2.5–5] days versus 2 [2,3] days (Table 2). Diabetes, hypertension, and renal failure were more prevalent among patients with common severe medical complications (Table 3).

**Table 2:** Demographic information of patients without and with common medical complications.

	No Common Complication	With Common Complication	
	Count (%) or Median (IQR)	Count (%) or Median (IQR)	P value
Total (N)	11224	68	
Age	64.0 (57, 71.5)	75.6 (63.1, 81.6)	< .0001
BMI	28.1 (24.8, 32.1)	28.0 (25.1, 33.3)	0.1976
Sex			0.1407
Female	5955 (53.1)	30 (44.1)	
Male	5268 (46.9)	38 (55.9)	
Race (if white)			0.0005
Missing	159 (1.4)	3 (4.4)	
No	1223 (10.9)	16 (23.5)	
Yes	9842 (87.7)	49 (72.1)	
Procedure			0.8273
Posterior	9296 (82.8)	57 (83.8)	
Anterior	1928 (17.2)	11 (16.2)	
Anesthetic Type			0.1383
Spinal	3584 (31.9)	16 (23.5)	
Epidural/CSE	7640 (68.1)	52 (76.5)	
Patient Class			0.1224
Ambulatory Surgery	105 (0.9)	0 (0)	
Inpatient	30 (0.3)	1 (1.5)	
Inpatient Surgery Admit	11089 (98.8)	67 (98.5)	
Surgeon Volume Per year			0.6163
0–55	683 (6.1)	4 (5.9)	
55–142	3613 (32.2)	25 (36.8)	
>142	6928 (61.7)	39 (57.4)	
Anesthesiologist Role			0.0007
Anesthesiologist Only	7856 (70)	55 (80.9)	
Anesthesiologist + Fellow	851 (7.6)	3 (4.4)	
Anesthesiologist + Resident	556 (5)	3 (4.4)	
Anesthesiologist + CRNA	1909 (17)	7 (10.3)	
Anesthesiologist + Multiple	52 (0.5)	0 (0)	
ASA			< .0001
ASA Level I&II	9254 (82.4)	37 (54.4)	
ASA Level III	1970 (17.6)	31 (45.6)	
Surgery Length (hr)	2.5 (2.3, 2.8)	2.6 (2.3, 3)	0.4796
Anesthesia Length (hr)	2.3 (2, 2.6)	2.4 (2.1, 2.8)	0.0849
Length of stay (day)	2 (2, 3)	3 (2.5, 5)	< .0001

Common complication is defined as any of the events happened from the day of surgery to discharge: Myocardial Infarction, stroke, or acute kidney injury;

IQR: Interquartile range; BMI: Body mass index; CRNA: Certified registered nurse anesthetists; CSE: Combined spinal epidural; ASA: American Society of Anesthesiologist Classification.

**Table 3:** Comorbidity of patients without and with common medical complications.

	No Common Complication	With Common Complication	
	Count (%)	Count (%)	P value
Total (N)	11224	68	
Any Comorbidity	7014 (62.4)	46 (90.2)	< .0001
Alcohol Abuse	61 (0.5)	0 (0)	>0.999
Anemia	315 (2.8)	6 (8.8)	0.0126
Rheumatoid arthritis	376 (3.3)	1 (1.5)	0.7293
Congestive heart failure	65 (0.6)	0(0)	>0.999
Chronic pulmonary disease	961 (8.6)	8 (11.8)	0.3795
Coagulation deficiency	170 (1.5)	2 (2.9)	0.2775
Depression	944 (8.4)	4 (5.9)	0.6588
Diabetes	614 (5.5)	12 (17.6)	0.0003
Drug Abuse	30 (0.3)	0 (0)	>0.999
Hypertension	3900 (34.7)	46 (67.6)	< .0001
Hypothyroidism	1086 (9.7)	11 (16.2)	0.0951
Liver disease	111 (1)	1 (1.5)	0.4933
Lymphoma	73 (0.6)	0 (0)	>0.999
Fluid and electrolyte disorders	412 (3.7)	2 (2.9)	>0.999
Metastatic cancer	205 (1.8)	0 (0)	0.6374
Other neurological disorders	206 (1.8)	4 (5.9)	0.0376
Paralysis	11 (0.1)	0 (0)	>0.999
Peripheral Vascular disease	191 (1.7)	2 (2.9)	0.3243
Psychoses	75 (0.7)	1 (1.5)	0.3691
Renal failure	196 (1.7)	10 (14.7)	< .0001
Solid tumor without metastasis	643 (5.7)	4 (5.9)	0.7961
Chronic peptic ulcer disease	189 (1.7)	0 (0)	0.6317
Valvular disease	460 (4.1)	4 (5.9)	0.3628
Sleep apnea	814 (7.3)	7 (10.3)	0.3425
Obesity	1110 (9.9)	9 (13.2)	0.3129
Pulmonary circulation disorders	62 (0.6)	2 (2.9)	0.0567
AIDS	48 (0.4)	1 (1.5)	0.2567
Weight Loss	17 (0.2)	0 (0)	>0.999

Note: Comorbidity variables included all major Elixhauser comorbidity conditions, and obstructive sleep apnea (OSA).

**Fig 2:**
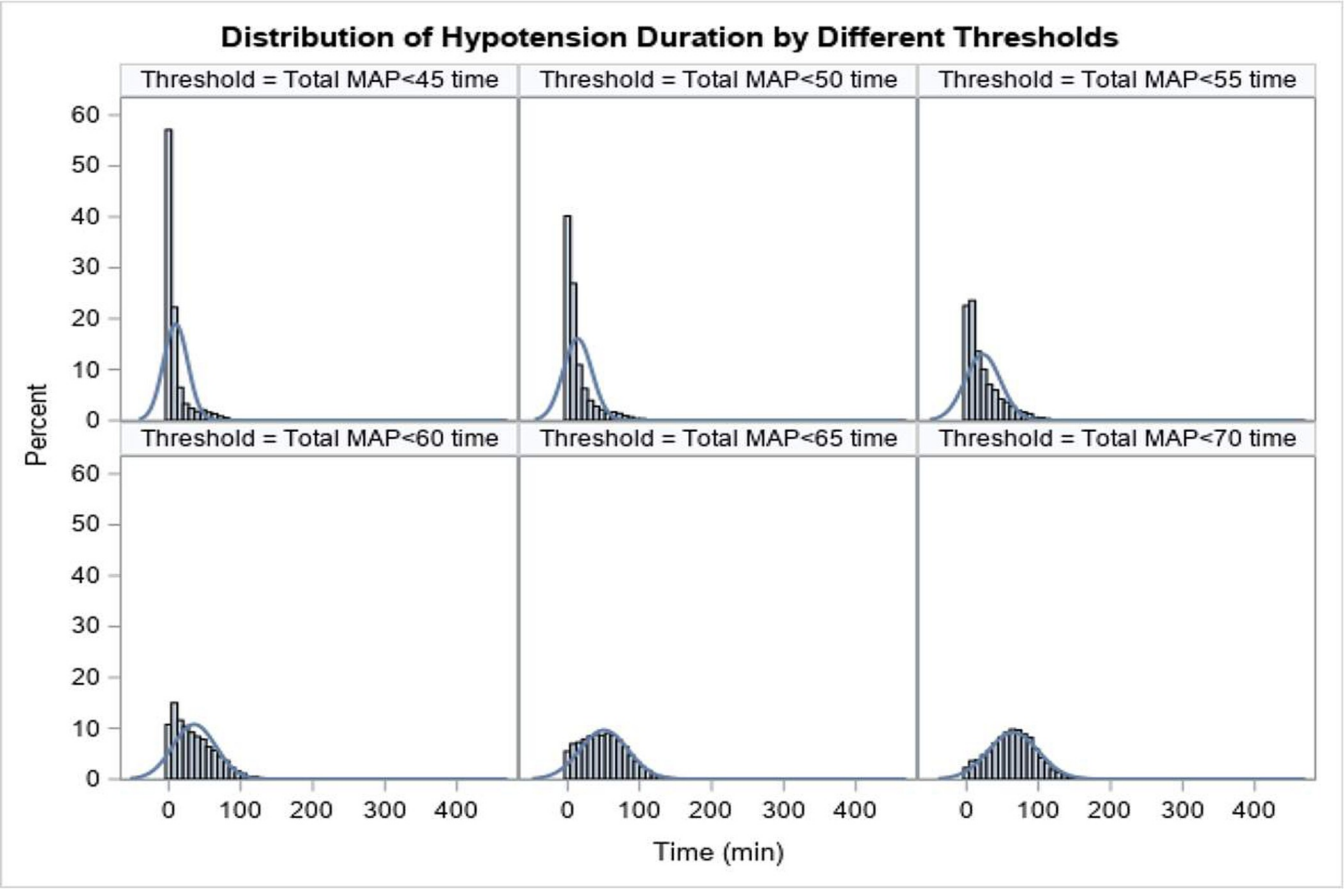
Distribution of hypotension duration by different thresholds. Thresholds were set at 45, 50, 55, 60, 65, and 70 mmHg. X-axis is duration in minutes, Y-axis is percentage of patients with hypotension minutes out of total patients.

The extent and duration of IOCH were generally limited to the essential portion of the surgery. There were 26.7%, 49.0%, 70.2%, and 84.6% of patients experienced MAP<45, 50, 55, and 60 mmHg respectively. The median duration of these IOCH episodes were 3 [1, 9], 6 [2, 17], 14 [4, 34], and 30 [11, 55] minutes respectively (Fig 2). Comparing patients with and without perioperative complication, there were no significant differences in the duration of IOCH at any of these thresholds.

In the regression analysis with composite common severe complications as dependent variable, MAP threshold of 60 mmHg applied as an example, and after adjusting for covariables, the OR of composite common severe medical complications was not significantly associated with duration of IOCH (OR 1.00 [0.99, 1.01]). The adjusted odds ratios for complications in older patients (OR, 1.08; CI, 1.05–1.10), ASA Level III patients (OR, 2.75; CI, 1.67–4.51) were significantly increased. White patients exhibited lower odds ratio comparing to non-white race (OR 0.29; CI 0.16–0.51). No other covariables had significant effects on the adjusted odds of composite common severe complications (Table 4).

**Table 4:** Multivariate logistic regression.

Effect	Odds Ratio (CI)	p value
Intercept		
Age	1.08 (1.05, 1.10)	< .0001
ASA (Level III vs Level I&II)	2.75 (1.67, 4.51)	< .0001
Block Type (Epidural+ Combined spinal epidural vs Spinal)	1.27 (0.71, 2.27)	0.4124
Race (white vs non-white)	0.29 (0.16, 0.51)	< .0001
Duration of MAP<60 (min)	1.00 (0.99, 1.01)	0.7275

Receiver Operating Characteristic (ROC) = 0.78

An area under the curve of 0.78 suggested the logistic regression model is effective in distinguishing patients with common severe complications from the ones with no complications. Given the small number of common severe complication events, the number of covariables included in the multiple logistic regression model is limited to avoid overfitting. We also conducted sensitivity analysis with additional independent variables, which did not contradict the results of regression. Therefore, the final model only included ASA classification to represent comorbidity. We also conducted sensitivity analysis on patients with myocardial infarction and stroke as the complication group, and found similar pattern of significance (OR 1.00 [0.99, 1.01]). Similarly, we also conducted sensitivity analysis with the 3 mortality patients included in the complication group, and the results were similar (OR 0.99 [0.98, 1.01]).

**Fig 3:**
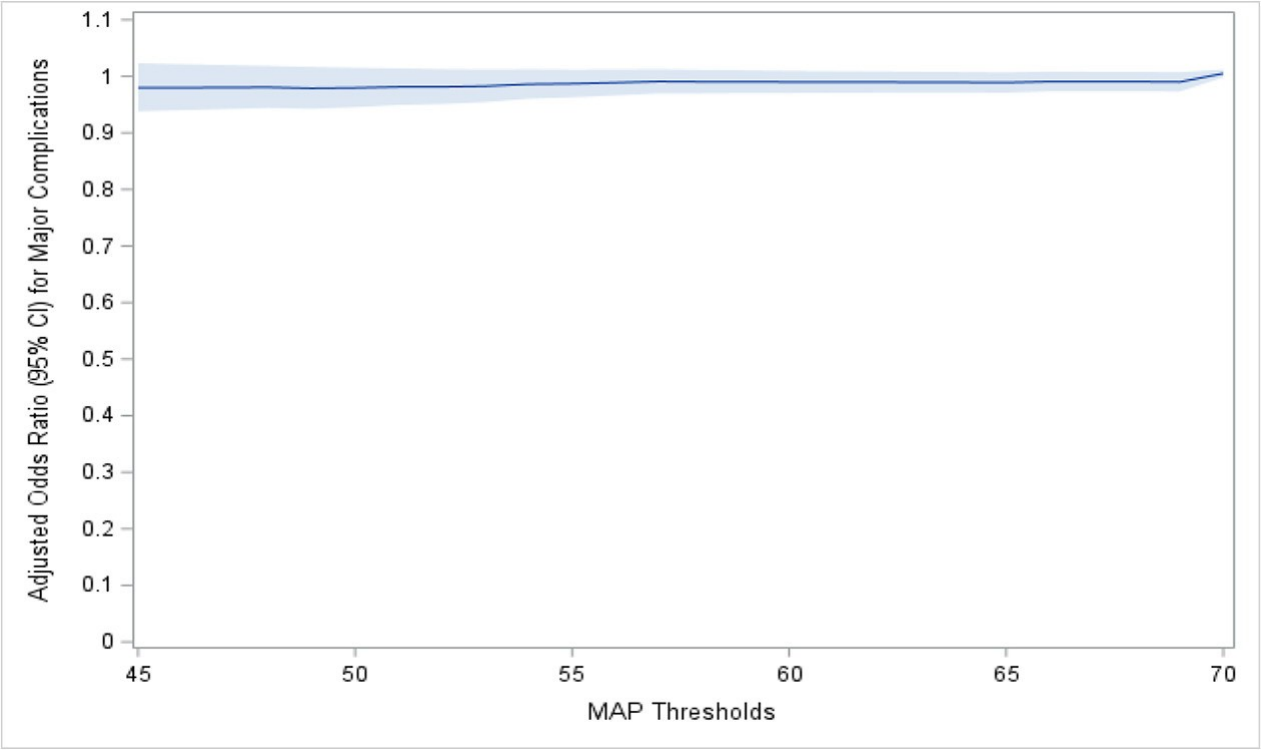
Odds ratio of common complications across various MAP cutoff thresholds. Odds ratio was calculated via logistics regression analyses with MAP ranging from 45 to 70 mmHg at 1 mmHg interval.

In subsequent serial logistics regression analyses with MAP threshold ranging from 45 to 70 mmHg at 1 mmHg interval, the duration of hypotension at various MAP thresholds did not increase the odds of common severe complications across various MAP cutoff thresholds from 45 to 70 mmHg (Fig 3).

## Discussion

In our study of 11,292 patients undergoing elective THA under neuraxial anesthesia with IOCH ranging from 45 to 70 mmHg, we observed no association between the level of IOCH and the likelihood of common severe complications, including myocardial infarction, stroke, and/or acute kidney injury. To our knowledge, our study is the largest to date assessing the effect of IOCH on the risk of common severe medical complications.

IOCH during arthroplasty surgery under neuraxial anesthesia differs from the general concept of intraoperative uncontrolled hypotension. With the use of vasoactive support, as is standard in our practice, cardiac output and thus perfusion can be maintained even in the setting of low blood pressures [11]. Uncontrolled hypotension, in contrast, can result from intraoperative morbid events or can be the cause of morbidity via hypoperfusion. Thus even the relationship between uncontrolled hypotension and morbidity and mortality remains poorly defined [17].

There is not a clear definition of what constitutes intraoperative hypotension [8]. There are, however, two commonly used and distinct approaches to defining hypotension. One uses an absolute blood pressure value, such as systolic blood pressure less than 80 to 100 mmHg or MAP values less than 60 mm Hg. Another approach uses the relative decrease of blood pressure in relation to the patient’s baseline with a 20–30% drop generally considered to be significant. The current consensus is that either definition is effective in providing valuable information about organ system perfusion [18]. In this study we elected to use absolute MAP values, in part because immediate pre-induction blood pressure values may not represent an accurate MAP baseline.

A number of large observational analyses have associated intraoperative MAPs below certain thresholds with higher odds of myocardial injury and mortality. The MAP thresholds used in the studies were <75 mmHg [7], <65 mmHg [18], <60 mmHg [19], <55 mmHg [9], and <50 mmHg [20]. It has been argued that the duration of hypotension can correlate with poor outcomes [21]. However, these observed hypotension events were not actively controlled and were confounded by instances of intraoperative hemodynamic instability related to physiologic derangements.

Futier et al. [22] conducted a randomized multicenter clinical trial of patients undergoing abdominal surgery; and targeting systolic blood pressure within 10% of baseline vs. 40% of baseline or 80 mmHg. The authors concluded that maintaining blood pressure close to baseline reduced the odds of organ dysfunction (OR 0.66, 95% CI 0.52~0.84). This study provided important evidence in regards to intraoperative hypotension. However, all these patients received general anesthesia and there was no support of cardiac output during hypotensive periods. Moreover, these passive hypotensive episodes were likely associated with detrimental events that may have contributed to the observed postoperative organ dysfunction. Under such circumstances, uncontrolled hypotension should be actively managed.

IOCH has been in practice for decades and has been predominantly used in specialized institutions focusing on neurosurgery and orthopedic surgery. During joint arthroplasty surgery, we achieve IOCH via a combination of neuraxial anesthesia and a vasoactive pressor agent to achieve and maintain a target blood pressure. Such an approach maintains cardiac output and blood flow to organ systems in a low pressure, low resistance system using epinephrine infusions in the range of 1–4 mcg/minute [11].

Many studies support the practice of IOCH [23–25]. Most of these studies were published in 1960s and 1970s with smaller sample size. More recently, investigators in our institution studied 2431 primary THA patients, and showed that there was no association between IOCH and postoperative acute kidney injury [24]. Our current study utilizing data from an electronic medical record encompassing a larger cohort provides further evidence in support of the safety of using IOCH combined with neuraxial anesthesia. While it has been well established that prolonged uncontrolled hypotension can be detrimental to major organ systems, controlled hypotension likely provides more benefits than risks to patients in certain surgical contexts. These potential benefits include less surgical bleeding with better surgical exposure, shorter surgical time, and less incidence of deep vein thrombosis/pulmonary embolism.

This study is not without limitations. First, it is a retrospective review of data from a single, specialized institution and for a single surgical procedure. This may limit generalizability of our conclusion to other patient populations, practice conditions and/or surgical procedures. Second, we only included patients who received neuraxial anesthesia as the small number who received general anesthesia have not followed the standard of care pathway in our institution and the physiology of hypotension under general anesthesia is distinct from that under neuraxial. Third, although IOCH is standard practice in our institution during primary THA surgery, we could not definitively distinguish between controlled versus inadvertent events in this database. However, a significant incidence of inadvertent hypotension would be expected to increase the rate of adverse outcomes and this was not observed. Fourth, this retrospective study could not account for variation in specific blood pressure targets between different anesthesiologists and among different patients. It must be assumed that surgeons/anesthesiologist teams are balancing concerns regarding patient comorbidities with conditions on the surgical field when determining targets for IOCH. Fifth, there were only sixty eight patients with common severe complications (0.60%) among 11,292 patients. Such low frequency of events limited our ability to analyze other potential interesting confounders. However, we believe that such low incidence of events with our practice is a testimony that IOCH, as practiced in our institution, does not increase the risk of common severe complications. Sixth, duration of IOCH varies across our study cohort. We could not determine whether the durations were all intentional and necessary. It is possible that brief periods of hypotension serve as ischemic preconditioning that protects against subsequent ischemia. Finally, patients were not followed after discharge for the development of common severe complications other than 30-day mortality. It is reasonable to believe that as patients met discharge criteria, the potential impact of IOCH on the development of complications after discharge would be negligible.

## Conclusions

Our study of 11,292 primary THA patients from a single institution showed that moderate duration of IOCH (as justified by the clinical situation) across various MAP thresholds (ranging from 45 to 70 mmHg) was not associated with increased odds of common severe in-hospital medical complications, including myocardial infarction, stroke, and/or acute kidney injury. IOCH is a distinct entity from passive, uncontrolled, and inadvertent hypotension and neuraxial-based IOCH could be considered and practiced for a reasonable duration when there are clear clinical indications.
